# Synthetic thermoresponsive scaffolds for the expansion and differentiation of human pluripotent stem cells into cardiomyocytes

**DOI:** 10.1039/d5ra04674b

**Published:** 2025-09-02

**Authors:** Luis F. Arrieta-Viana, Janet Mendez-Vega, Madeline Torres-Lugo

**Affiliations:** a Department of Chemical Engineering, University of Puerto Rico-Mayagüez Mayagüez Puerto Rico USA madeline.torres6@upr.edu

## Abstract

The advancement of regenerative medicine requires robust, reproducible, and scalable platforms for the expansion and differentiation of human pluripotent stem cells (hPSCs) into specialized cells, such as cardiomyocytes. While current natural matrices like Matrigel™ suffer from batch-to-batch variability and limited tunability, synthetic scaffolds with controllable biochemical and mechanical properties could provide superior platforms for maintaining stem cell pluripotency and directing efficient cardiac differentiation. Here, we report the development and evaluation of a customizable thermoresponsive terpolymer composed of *N*-isopropylacrylamide (NiPAAm), vinylphenylboronic acid (VPBA), and polyethylene glycol monomethyl ether monomethacrylate (PEGMMA) synthesized *via* free-radical polymerization as a synthetic matrix for human hPSC culture. This terpolymer exhibited optimal properties, including tunable stiffness, transparency, and thermoresponsiveness, facilitating non-invasive cell harvesting and downstream applications, characterized by flow cytometry, immunofluorescence, and gene expression analysis. In both two-dimensional (2D) and three-dimensional (3D) culture conditions, the terpolymer effectively supported the maintenance of pluripotency and promoted robust proliferation of human induced pluripotent stem cells (hiPSCs) and human embryonic stem cells (hESCs). Furthermore, incorporation of bioactive molecules into the synthetic matrix such as RGD peptides, vitronectin, and fibronectin significantly enhanced cell expansion, aggregation, and cardiac differentiation efficiency. Differentiated cardiomyocytes exhibited statistically significant increases in the expression of cardiac-specific markers (cTnT and cTnI) reaching ∼65% and ∼25% respectively, compared to cells cultured on traditional matrices like Matrigel™, Cultrex™ and synthetic VitroGel™. These findings demonstrated the potential of this synthetic terpolymer scaffold as a reliable and scalable alternative for hPSC culture and differentiation, with significant implications for regenerative medicine, drug screening, and cardiac disease modeling.

## Abstract 2

The advancement of regenerative medicine requires robust, reproducible, and scalable platforms for the expansion and differentiation of human pluripotent stem cells (hPSCs) into specialized cells, such as cardiomyocytes.

## Graphical abstract

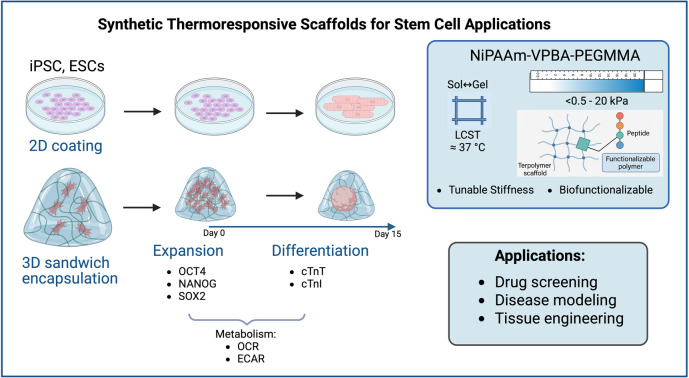

## 1. Introduction

Tissue engineering and advanced cell culture platforms are essential tools in biomedical research and biomanufacturing, enabling precise control over cellular behavior and differentiation *in vitro*.^1,2^ Among these platforms, human pluripotent stem cells (hPSCs)—including human induced pluripotent stem cells (hiPSCs) and human embryonic stem cells (hESCs)—are particularly valuable due to their ability to differentiate into virtually any cell type.^3^ This unique potential makes hPSCs indispensable in regenerative medicine, where they can be directed to generate specialized cells, such as cardiomyocytes, neurons, and hepatocytes for therapeutic applications.^4–6^ Cardiomyocytes, the heart's primary contractile cells, are responsible for pumping blood throughout the body. However, cardiovascular diseases such as myocardial infarction (MI) lead to the irreversible loss of these cells, as adult cardiomyocytes lack the capacity to regenerate effectively.^7,8^ This creates an urgent need for *in vitro* systems capable of reliably producing functional cardiomyocytes for regenerative therapies and disease modeling. Despite their promise, differentiating hPSCs into mature cardiomyocytes remains challenging, as the resulting cells often exhibit fetal-like phenotypes with limited contractility and immature electrophysiological properties, reducing their therapeutic potential.^9–11^ In addition to their role in cardiac regeneration, hPSCs are critical for applications in drug discovery, disease modeling, and toxicity testing offering personalized treatment options when derived from the patient's own cells.^12^

Nevertheless, harnessing the full potential of hPSCs requires culture systems that closely replicate the dynamic and complex *in vivo* microenvironments, supporting hPSC expansion, pluripotency, and efficient lineage-specific differentiation state.^13,14^ The extracellular matrix (ECM) plays a key role in guiding cell behavior by providing structural support and biochemical signals.^15,16^*In vivo*, stem cells are maintained in specialized niches, where interactions between ECM components and cell-surface receptors tightly regulate self-renewal and differentiation.^17^ Current matrices, such as Matrigel™—a basement membrane extract (BME) derived from Engelbreth–Holm–Swarm (EHS) mouse tumors—consist of laminin, collagen IV, entactin, proteoglycans, and various growth factors.^18–20^ Cultrex™, another BME derived from the same tumor source, provides a similar composition and is often used as a defined alternative to Matrigel™.^21,22^ However, these xenogeneic matrixes present significant disadvantages, including batch variability, undefined composition, and limited scalability, which decrease their reproducibility and large-scale application.^20^ Additionally, these natural matrices lack the flexibility needed to adjust to different mechanical and biochemical cellular environments, which limits their effectiveness across various biomedical applications.

To address these challenges, synthetic polymer scaffolds have emerged as promising alternatives, offering precise control over key properties like stiffness, ligand presentation, and biochemical functionality.^23–27^ These synthetic platforms address many of the limitations associated with natural matrices.^28^ Unlike animal-derived products, synthetic scaffolds ensure consistent quality across batches, reducing variability and enhanced reproducibility, an essential requirement for both experimental standardization and clinical applications. Additionally, synthetic scaffolds can support both 2D^29,30^ and 3D cultures,^31–35^ providing greater flexibility for various biomedical applications, from basic research to tissue engineering. They can also be tailored to incorporate specific bioactive motifs, such as RGD peptides,^36,37^ vitronectin,^38,39^ and fibronectin,^40^ promoting cell adhesion, proliferation, and differentiation. This customization offers a potential solution to the common issue of immature phenotypes in iPSC-derived cardiomyocytes. Furthermore, synthetic scaffolds mitigate immunogenic risks and ethical concerns by eliminating the need for animal-based materials.^41^ Their scalability and cost-effectiveness make them particularly well-suited for large-scale applications in tissue engineering, regenerative medicine, and drug discovery.

One of the earliest efforts in the development of synthetic scaffolds involved studies of peptide-acrylate surfaces (PAS) functionalized with bone sialoprotein and vitronectin peptides.^42^ These matrices supported the long-term maintenance of hESCs in chemically defined, xeno-free conditions, enabling cardiomyocyte differentiation and scalability. Despite these advancements, PAS faced challenges in achieving fully mature cardiomyocyte phenotypes and required extensive optimization to replicate the ECM environment. Building on this, poly(ethylene glycol) (PEG)-based hydrogels modified with integrin-binding peptides such as RGDS and YIGSR were proposed to facilitate iPSC adhesion and differentiation into neural progenitors.^43^ While effective, these materials had limitations in replicating the intricate mechanical and biochemical signals of the native ECM. Similarly, the use of poly[2-(methacryloyloxy) ethyl dimethyl-(3-sulfopropyl)ammonium hydroxide] (PMEDSAH),^44^ a zwitterionic hydrogel, for feeder-free culture of transgene-free iPSCs was investigated by Villa-Diaz *et al.* This system maintained pluripotency and genomic stability for nine months, yet its broader applicability in directing specific lineage differentiation was limited. Additional studies have advanced synthetic scaffolds to better mimic native ECM properties. For example, methacrylated gelatin (GelMA)^45,46^ hydrogels have shown promise in supporting hPSC proliferation and guiding differentiation through tunable stiffness and bioactivity. However, their inability to efficiently produce mature cardiomyocytes and their lack of scalability for therapeutic use remain significant challenges. Moreover, dynamic covalent hydrogels,^47,48^ capable of real-time modulation of cell environments, have enabled better control over stem cell fate decisions. However, the interplay between dynamic mechanical properties and biochemical signals often requires further refinement for achieving consistent lineage-specific differentiation.

Building on these developments, our laboratory previously designed a customizable thermo-responsive terpolymer composed of NiPAAm, VPBA, and PEGMMA. This terpolymer offers a unique combination of tunable mechanical properties, biofunctionalization, and thermo-responsiveness. Its thermo-responsive behavior enables non-invasive cell recovery, thereby facilitating downstream applications. This platform has been optimized for culturing various cell lines, including SKOV-3 ovarian cancer cells, NIH-3T3 fibroblasts, Leukemia Jurkat T cells, Pan-T cells, CAR-T cells and patient-derived glioblastoma cells.^49–51^ Previous characterization demonstrated that the terpolymer formulations exhibited physiologically relevant lower critical solution temperatures (LCST) near 37 °C and tunable mechanical stiffness ranging from 0.5 to 18 kPa, enabling customization for different cellular applications. The biocompatible platform also showed capability for functionalization with bioactive molecules, providing opportunities for enhanced cell–matrix interactions.

The present study aimed to evaluate whether these synthetic terpolymer scaffolds could serve as effective platforms for hPSC and ESC culture and cardiomyocyte differentiation. We systematically investigated the performance of terpolymers functionalized with bioactive molecules including RGD peptides, vitronectin, and fibronectin compared to traditional natural matrices like Matrigel™ and Cultrex™, as well as commercial synthetic alternatives such as VitroGel™. The research examined how the incorporation of these ECM-derived molecules affects pluripotency maintenance through assessment of key transcription factors and how controlled mechanical properties influence cardiac lineage. Additionally, we explored the versatility of terpolymer blending to achieve tunable stiffness ranges suitable for cardiac applications. This comprehensive evaluation sought to determine the potential of our synthetic platform as a scalable, reproducible alternative to existing matrices while providing enhanced control over cell–matrix interactions to improve cardiac differentiation efficiency in both 2D and 3D culture systems.

## 2. Materials and methods

### 2.1. Thermos-responsive scaffolds synthesis and preparation

The protocol for synthesizing and preparing the thermo-responsive terpolymer scaffold has been described elsewhere.^49^ Briefly, the terpolymers were synthesized by free-radical polymerization using *N*-isopropylacrylamide (NiPAAm), 4-vinylphenylboronic acid (VPBA) obtained from (Sigma Aldrich, St. Louis, MO, USA), and poly(ethylene glycol) monomethyl ether methacrylate (PEGMMA) from (Polysciences, Inc. Valley Rd, Warrington, PA, USA). The molar ratios of the monomers were varied to optimize properties like lower critical solution temperature (LCST) and mechanical stiffness. The polymerization was initiated with 2,2′-azobis(2-methylpropionitrile) (AIBN) (Sigma Aldrich, St. Louis, MO, USA) at 65 °C in anhydrous ethanol (Sigma Aldrich, St. Louis, MO, USA) for 24 hours. The mixture was continuously stirred at 150 rpm with nitrogen (Praxair, Guaynabo, Puerto Rico) flow maintained throughout the reaction. Following synthesis, the polymers were precipitated using petroleum ether (Sigma Aldrich, St. Louis, MO, USA), dried and ground into powder for storage and characterization.

After characterization, the polymers were washed by dissolving in cold deionized (DI) water, followed by thermo-precipitation at 50 °C. The precipitated samples were re-dissolved in cold DI water, dried, and ground into powder. For sterilization, the terpolymer was dissolved in cold DI water to form a 40 wt% solution and filtered using a hydrophilic PES membrane (Millipore Sigma, St. Louis, MO, USA) filter in a sterile laminar flow hood. The sterilized solution was stored in a glass vial for later use in cell culture.

### 2.2. Scaffold characterization

The thermal and mechanical properties, focusing on the low critical solution temperature (LCST) and storage modulus were assessed by oscillatory strain using a modular compact rheometer rheometer MCR 302 (Anton Paar, GmbH, Graz, Austria). Polymer solutions at 15 wt% were prepared in deionized water and loaded onto a parallel-plate geometry with a 25 mm diameter and a 0.5 mm gap setting. The temperature was gradually increased from 10 °C to 60 °C at a rate of 3 °C min^−1^ to monitor the transition from the sol (liquid) state to the gel state, indicating the LCST. The storage modulus (*G*′) was recorded as a function of temperature to determine the start of gelation and the LCST, noted at the temperature where *G*′ sharply increased. Stiffness was analyzed at 37 °C, simulating physiological conditions, with the *G*′ measured at a constant frequency of 5 Hz and 2% strain.

### 2.3. Cell culture

#### 2.3.1. Human pluripotent stem cell lines

The hiPSC lines WTC-11 and hESC lines H9 (WiCell, Madison, WI, USA) were maintained using the feeder-free method. Cells were cultured on Cultrex basement membrane extracts (Cultrex™) (R&D Systems, Minneapolis, USA)—a defined ECM substitute derived from EHS mouse sarcoma and used as an alternative to Matrigel™^21^—coated 6-well plates using mTeSR-1 (StemCell Technologies, Vancouver, Canada) medium under feeder-free conditions, following a defined procedure based on Ludwig *et al.*^52^ Cells were passaged upon reaching 70–80% confluency using Versene (Fisher Scientific, New Jersey, USA) to gently dissociate cells and maintain pluripotency. Cultures were incubated at 37 °C with 5% CO_2_, with daily medium changes.

#### 2.3.2. Differentiation of hiPSC and hESC into cardiomyocytes

The differentiation of human iPSCs (WTC-11) and hESCs (H9) into cardiomyocytes was performed following the Lian *et al.*^53^ protocol with optimizations for CHIR99021 (Tocris, Bristol, United Kingdom) concentration, and cell density. Due to variability between CHIR99021 lots, concentrations ranging from 5 to 12 μM were tested alongside seeding densities of 50 000 to 150 000 cells per cm^2^. Cells at 70–80% confluency were replated on Cultrex™-coated plates, with ROCK inhibitor in mTeSR1 medium. Differentiation was initiated by switching to RPMI1640 (Sigma Aldrich, St. Louis, Missouri USA) with B27-(Gibco, Waltham, USA) and CHIR99021 on day 0. After 48 hours, Wnt inhibition was induced with IWP2 (Tocris, Bristol, United Kingdom), followed by media changes with RPMI1640 and B27-supplements on day 4. RPMI1640 and B27+ (Gibco, Waltham, USA) supplements were added at days 6, and 8, continuing every three days until day 15. Optimized conditions ensured consistent cardiomyocyte differentiation for subsequent experiments.

#### 2.3.3. Two and three-dimensional cell culture

##### 2.3.3.1 Coating method

To prepare plates for cell culture in a 2D condition, a 15 wt% layer of previously sterilized terpolymer solution was applied at 360 μL cm^−2^. The solution was evenly spread by gently tilting the plates within a Laminar Flow Hood (LFH). Plates were incubated at 37 °C with 5% CO_2_ for at least two hours to allow for terpolymer gelation. For plates without peptides, the supernatant was aspirated after gelation, and cells were added at 100 000 cell per cm^2^ directly onto the terpolymer-coated surface. The plates were gently agitated to evenly distribute the cells. For peptide-coated plates, a solution containing RGD, RGDS, vitronectin, or fibronectin peptides was added after initial gelation. The plates were incubated for an additional two hours before the supernatant was removed and cells were seeded, followed by gentle agitation for even distribution before incubation under standard culture conditions. Cultrex™ coatings were performed at 100 μL cm^−2^ and used as a control for this method.

##### 2.3.3.2 Sandwich method

This technique was used to encapsulate cells within a 3D polymer matrix. For this purpose, a base layer of terpolymer was added and spread uniformly as described in the Coating method. Cells were then mixed with terpolymer solution for even distribution and deposited over the base layer at 15 wt% in culture media. When using functionalized terpolymers, a peptide solution (RGD, RGDS, vitronectin, or fibronectin) was added to the terpolymer mixture. Cells were incubated at 37 °C with 5% CO_2_ and the culture media was replaced every 24 hours. For 3D culture controls, the xeno-free VitroGel™ system was used. Cells were suspended in mTeSR-1 medium at the same cell concentration as that used in the terpolymer formulations. After 10–20 minutes at room temperature for gel formation, additional culture medium was added to stabilize the scaffold, and the plates were incubated at 37 °C with 5% CO_2_.

### 2.4. Cell harvesting

hiPSC and hESC were harvested from Cultrex™ at 70–80% confluency, and cardiomyocytes were harvested 15 days post-differentiation under 3D conditions. For harvesting, 500 μL of cooled (4 °C) 20 mM fructose solution was added and incubated for 10 minutes as described before.^49^ The contents were transferred to a centrifuge tube and spun at 220 rcf for 5 minutes. The supernatant was discarded, and cell pellets were treated with Accutase (Innovative Cell Technologies, San Diego, USA) for 5 minutes, followed by centrifugation at 200 rcf for 5 minutes. After removing the Accutase, cells were resuspended in 1 mL DMEM for analysis.

For cells cultured in VitroGel™, cells were washed with PBS and treated with 0.5 mL of VitroGel™ Organoid Recovery Solution (The Well, North Brunswick, USA) for 5 minutes before being transferred to a 15 mL conical tube with 1 mL DMEM. The cells were centrifuged at 200 rcf for 5 minutes to complete harvesting.

### 2.5. Flow cytometry analysis

Flow cytometry was performed using an Accuri C6 Plus Flow Cytometer (BD Biosciences, San Jose, CA, USA) to verify pluripotency and cardiomyocyte differentiation.^54^ For pluripotency assessment, hiPSCs and hESCs were analyzed for Oct4 (Cell Signaling, Boston, USA) and Nanog (Cell Signaling, Boston, USA). Cardiomyocytes at day 15 of differentiation were analyzed for cardiac Troponin T (cTnT, Abcam, Connecticut, USA) and cardiac Troponin I (cTnI, Abcam, Connecticut, USA) as markers of differentiation and maturation. Cells were first washed with PBS, dissociated with Accutase (5 minutes), and centrifuged at 220 rcf for 5 minutes. Cell counts were determined using a hemocytometer, and samples were prepared at 200 000 cells per mL. Cells were then fixed with 1% (v/v) paraformaldehyde in PBS and permeabilized with 90% (v/v) methanol (Sigma Aldrich, St. Louis, MO, USA). After washing with a solution of 1% (w/v) BSA and 0.5% (v/v) Triton X-100 (Sigma Aldrich, St. Louis, MO, USA) prepared in PBS, cells were incubated overnight at 4 °C in the dark with primary antibodies diluted 1 : 200 in the same blocking/permeabilization buffer. Secondary antibodies were applied as follows: Alexa 488 (Invitrogen, California, USA) for Nanog and cTnT, and Alexa Fluor 647 (Thermo Fisher Scientific, Waltham, USA) for Oct4 and cTnI.

### 2.6. Immunofluorescence staining

Immunostaining was performed to evaluate pluripotency and cardiomyocyte differentiation using fluorescence microscopy with an Olympus IX83 confocal microscope (Center Valley, PA, USA). Human induced pluripotent stem cells and embryonic stem cells were washed three times with PBS, fixed with 4% paraformaldehyde for 15 minutes at room temperature, and permeabilized and blocked in PBS containing 0.5% Triton X-100 and 1% BSA for 1 hour to minimize nonspecific binding. Primary antibodies targeting Oct4 and Nanog as pluripotency markers or cTnT and cTnI as cardiomyocyte markers were diluted at 1 : 200 in the blocking solution and incubated with the cells overnight at 4 °C. After three PBS washes, Alexa Fluor 488 and Alexa Fluor 647 conjugated secondary antibodies were applied at a dilution of 1 : 2000 for 1 hour at room temperature in the dark.^55^

### 2.7. Metabolic potential

The metabolic activity of cells cultured on terpolymer was assessed using a Seahorse XF Analyzer (Agilent Technologies, Santa Clara, USA) to measure extracellular acidification rate (ECAR) and oxygen consumption rate (OCR).^56^ Initially, cells were plated at a density of 60 000 cells per well in a 24-well plate coated either with terpolymer or with Cultrex® (used as a control). After 72 hours, cells were dissociated with Accutase and reseeded into a Seahorse XF 24-well microplate (Agilent Technologies, Santa Clara, USA) coated with Cultrex®. The cells were cultured in mTeSR-1 medium for 24 hours to ensure proper adhesion and recovery. Prior to the assay, cells were washed with pre-warmed XF DMEM Medium (Agilent Technologies, Santa Clara, USA) and incubated in the assay medium at 37 °C without CO_2_ for one hour to equilibrate temperature and pH. The Seahorse XF Cell Mito Stress Test was then performed according to manufacturer's instructions to assess metabolic parameters. Following the Seahorse analysis, cells were stained with Hoechst (Thermo Fisher Scientific, New Jersey, USA) and observed under a Keyence microscope (Keyence Corporation of America, Itasca, USA) for cell counting.

### 2.8. RNA isolation and quantitative PCR

Total RNA was extracted using the PureLink RNA Mini Kit (Thermo Fisher Scientific, New Jersey, USA) following the manufacturer's protocol. RNA quantity was measured with a Qubit 4 fluorometer (Thermo Fisher Scientific, New Jersey, USA). Pluripotency of hiPSCs and hESCs was evaluated *via* quantitative real-time PCR (qRT-PCR) using TaqMan RNA probes specific for OCT4 (Hs04260361_g1), NANOG (Hs04399610_g1), and SOX2 (Hs00602736_S1). GAPDH (Hs99999905_m1) served as the housekeeping gene for normalization. For cardiomyocyte differentiation, gene expression of TNNi3 (Hs00165957_m1), TNNT2 (Hs00943911_m1), CACNA1C (Hs00167681_m1), and MYH6 (Hs01101425_m1) was assessed using the TaqMan RNA probe method. Relative gene expression was calculated using the 2^(−ΔΔ*Ct*)^ method, normalizing against GAPDH. qRT-PCR reactions were prepared using the TaqMan Gene Expression Master Mix, with each 20 μL reaction containing 2 μL RNA, 1 μL TaqMan RNA probe, 11.5 μL Master Mix, and 6.5 μL nuclease-free water. The qRT-PCR was performed on a Quantum Studio 3 Real-Time PCR System (Thermo Fisher Scientific, New Jersey, USA) with the following cycling parameters: 95 °C for 10 minutes, followed by 40 cycles of 95 °C for 15 seconds and 60 °C for 1 minute.

### 2.9. Statistical analysis

All statistical analyses were performed using GraphPad Prism version 10.0 (GraphPad, San Diego, CA, USA) for Windows. Data are presented as mean ± standard error of the mean (SEM) from at least triplicate experiments unless otherwise specified. Parametric tests including Student's *t*-test for two-group comparisons and one-way ANOVA followed by Turkey *post hoc* test for multiple comparisons were used. Relative gene expression was calculated using the 2^(−ΔΔ*Ct*)^ method with GAPDH as reference gene. Statistical significance was set at *p* < 0.05, with significance levels denoted as *p < 0.05, **p < 0.01, ***p < 0.001, ****p < 0.0001, ns = not significant.

## 3. Results and discussion

### 3.1. hPSCs expansion and maintenance on 2D thermoresponsive scaffolds

Based on comprehensive characterization of NiPAAm-*co*-4-VPBA-*co*-PEGMMA terpolymers previously reported by our group^49^ the combination of 2% 4-VPBA, 4% PEGMMA400, and 94% NiPAAm (designated as 2 : 4 : 94 P400) was selected for hPSC culture and differentiation studies. This formulation was previously characterized and shown to exhibit optimal properties including a lower critical solution temperature near physiological conditions (37.0 ± 0.63 °C), suitable mechanical stiffness (17.8 ± 2.5 kPa), and optical transparency for cell monitoring. The terpolymer previously demonstrated successful application in multiple cell culture systems,^49–51^ suggesting its potential as a synthetic alternative to traditional matrices for stem cell applications.

**Fig. 1:**
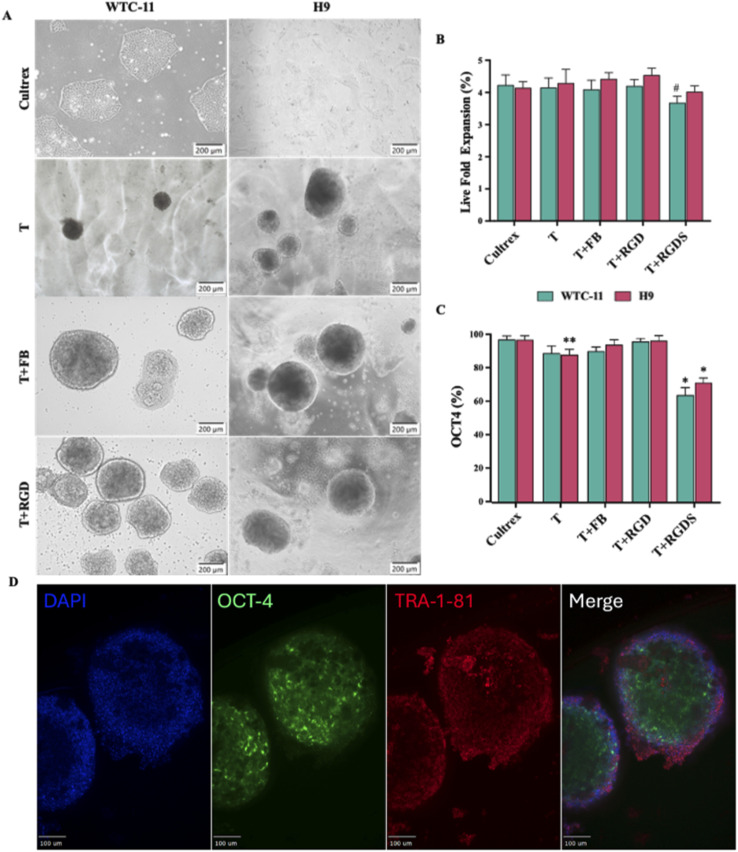
Impact of peptide incorporation on hPSC morphology and function. (A) Representative brightfield micrographs comparing WTC-11 and H9 hPSC cultures across different substrate conditions: Cultrex™, terpolymer alone (T), terpolymer with fibronectin (T + FB), and terpolymer with RGD peptide (T + RGD). Scale bars: 200 μm. (B) Cell proliferation measured as live fold expansion for both cell lines when cultured on 2 : 4 : 94 P400 terpolymer (15 wt%) modified with different bioactive molecules (FB, RGD, RGDS; 6 μg per well). (C) Maintenance of pluripotency assessed by OCT4 expression using flow cytometry under identical culture conditions. Data shown as mean ± SD with *n* = 5. Statistical significance: *p < 0.0001, **p = 0.081, #*p* = 0.0383 compared to Cultrex™. (D) Immunofluorescence images of WTC-11 hiPSCs cultured on 2 : 4 : 94 P400 terpolymer scaffolds (15 wt%) demonstrating expression of pluripotency markers OCT-4 (green) and TRA-1-81 (red), with nuclear counterstaining using DAPI (blue). Merged images confirm maintenance of stem cell identity. Scale bars: 100 μm.

Initial assessment of hPSCs behavior on the terpolymer scaffold concentrated on evaluating growth patterns, morphological characteristics, and maintenance of pluripotency markers in 2D culture. Cell proliferation was quantified using live fold expansion measurements to determine the scaffold's capacity to support robust cellular growth over time. WTC-11 and H9 cells were seeded at a density of 100 000 cells per cm^2^ on 24-well plates coated with 2 : 4 : 94 P400 terpolymer at 15 wt%. The coating was achieved by applying 360 μL cm^−2^ of terpolymer solution per well, which was selected for its optimal transparency and physiological gelation temperature. When culturing iPSCs (WTC-11) and ESCs (H9) on different surfaces, distinct differences in how the cells organized themselves was observed (Fig. 1A). On Cultrex™ surfaces, both cell lines formed the characteristic flat, spread-out colonies typical of 2D culture, consistent with previous reports.^19,57^ However, when grown on the 2 : 4 : 94 P400 terpolymer scaffold, cells adopted a more three-dimensional, spheroid-like appearance (Fig. 1A, compare T, T + FB, and T + RGD conditions with Cultrex control). This morphological transformation aligns with observations in previous studies where iPSCs cultured on nanofibrillar cellulose hydrogels similarly developed rounded morphologies while maintaining high OCT4 expression.^58^ This type of spheroid formation reflects the unique interactions between cells and synthetic surfaces. Previous research has shown that surface chemistry and polymer composition significantly influence how stem cells organize into colonies.^59^ The hydrophobic-hydrophilic balance created by the combination of NiPAAm, VPBA, and PEGMMA may create a microenvironment that favors three-dimensional aggregation over two-dimensional adhesion. Similar morphological transitions have been observed during the long-term culture of human pluripotent stem cells on other synthetic polymer surfaces, where cells spontaneously formed aggregates, while maintaining their undifferentiated state.^60,61^ These synthetic surfaces often lack the extensive protein-binding sites found in natural matrices, leading cells to preferentially interact with each other rather than with the substrate.^55,56^ Together, these findings suggest the proposed terpolymer provides biophysical and biochemical cues that resemble the three-dimensional architecture of the native stem cell niche, promoting spontaneous spheroid formation, while maintaining pluripotency.

### 3.2. Incorporation of cell-adhesive biomolecules enhances the functionality of 2D synthetic terpolymer matrices

To further enhance the biomimetic properties of the scaffolds, various ECM-derived molecules such as RGD, RGDS peptides, and fibronectin were incorporated. The rationale behind this approach was based on the crucial roles of these molecules in cell–matrix interactions within natural tissues.^62^ RGD and RGDS sequences are specifically recognized by cell surface integrins such as α5β1 and αvβ3,^36^ while fibronectin provides multiple binding sites that promote cell attachment and maintain pluripotency-related signaling pathways.^40^ Terpolymers were then enriched the with these bioactive molecules at 6 μg per well. Images revealed colony formation, particularly with RGD peptides and fibronectin, where cells formed notably larger and more compact spheroid structures (Fig. 1A).

**Fig. 2:**
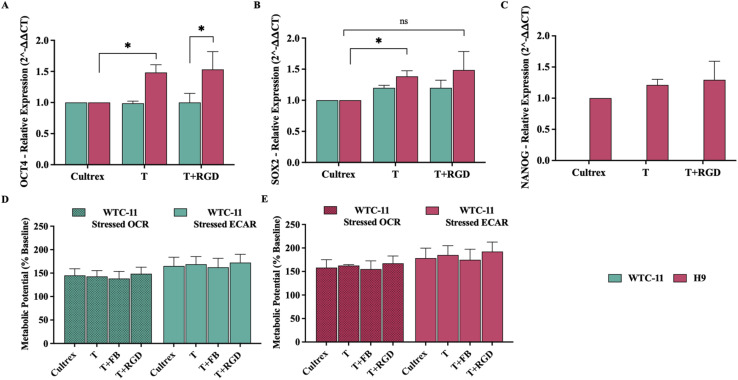
Gene expression profiles and metabolic activity of WTC-11 iPSCs and H9 hESCs cultured on the terpolymer (2 : 4 : 94 P400) with and without the addition of peptides (fibronectin (FB), RGD) at a concentration of 6 μg per well. (A) Relative expression of OCT4 assessed by qPCR for WTC-11 and H9 cells. (B) Relative expression of SOX2 assessed by qPCR for WTC-11 and H9 cells. (C) Relative expression of NANOG assessed by qPCR for H9 cells. (D) Metabolic potential (stressed OCR and ECAR) measured by Seahorse analysis for WTC-11 cells. (E) Metabolic potential (stressed OCR and ECAR) measured by Seahorse analysis for H9 cells. Cultrex™ was used as the control substrate for all comparisons. Statistical comparison between cell lines under T + RGD condition was performed to evaluate differential responses of iPSCs *versus* ESCs to the same scaffold modification. Statistical significance: *p < 0.03. Bars represent mean ± STDV (*n* = 3 for gene expression, *n* = 5 for metabolic potential assays).

More importantly, the improvements in colony morphology were accompanied by consistent cell proliferation. Both WTC-11 and H9 cells exhibited comparable live fold expansion rates under most conditions (Fig. 1B). A slight decrease in proliferation was only observed for WTC-11 cells cultured on T + RGDS, suggesting that this particular modification may be less optimal for supporting iPSC growth. These results are consistent with previous reports that demonstrated that stem cell responses to synthetic substrates can vary depending on integrin expression profiles across different cell lines.^55,63,64^ Flow cytometry analysis was employed to quantitatively assess the expression of key pluripotency markers, providing objective validation of stem cell identity maintenance. Similar to the expansion results, cells maintained their pluripotent state across most culture conditions, as evidenced by OCT4 expression levels (Fig. 1C) by flow cytometry. Both cell lines showed robust OCT4 expression (>95%) on Cultrex™. In the case of the unmodified terpolymer with H9 cells, OCT4 expression was slightly lower (88.43 ± 3.11%), but notably, the addition of RGD peptides and fibronectin to the terpolymer improved this expression to levels comparable to commercial matrices (91.5 ± 3.8% and 96.2 ± 2.4%). However, a significant reduction in OCT4 expression was observed with T + RGDS, particularly in WTC-11 cells, suggesting that this specific peptide sequence may adversely affect both proliferation and pluripotency maintenance. This finding aligns with studies showing that different integrin-binding motifs can have variable effects on pluripotent stem cell self-renewal pathways.^65^ For the case of immunostaining analysis, results clearly demonstrated robust nuclear expression of OCT-4 and surface expression of TRA-1-81 markers in hPSCs cultured on the 2 : 4 : 94 P400 terpolymer (Fig. 1D). These observations align closely with the flow cytometry results, further confirming high expression of key pluripotency markers comparable to established commercial matrices. Quantitative gene expression analyses for OCT4, SOX2, and NANOG *via* qPCR provided additional validation (Fig. 2A–C). For both cell lines, expression levels were comparable or even superior to Cultrex™, notably in the case of OCT4 for H9 cells, indicating enhanced pluripotency maintenance on the terpolymer scaffold. These gene expression profiles align with previous findings suggesting that synthetic scaffolds, when properly functionalized, can effectively support pluripotency by providing appropriate biochemical cues similar to traditional substrates.^44,55^

Metabolic profiling using Seahorse XF Analyzer was performed to evaluate cellular bioenergetics, as metabolic state serves as a sensitive indicator of cellular health and pluripotency status. Seahorse XF Analyzer measurements indicated comparable metabolic profiles (OCR and ECAR) for cells cultured on the terpolymer relative to Cultrex™ controls (Fig. 2D and E). For WTC-11 cells, OCR values of 142.8 ± 12.6% (T) and 148.7 ± 13.8% (T + RGD) were statistically equivalent to Cultrex™ control (145.0 ± 14.3%) as well as ECAR values (T: 168.9 ± 16.4%, T + RGD: 172.4 ± 17.8% *vs.* control: 165.2 ± 18.7%). H9 cells exhibited metabolic activity with OCR values of 162.7 ± 14.9% (T) and 167.4 ± 15.6% (T + RGD) compared to control (158.3 ± 16.8%), reflecting the characteristic metabolic of ESCs. Pluripotent stem cells typically exhibit a balanced metabolic state characterized by relatively high ECAR accompanied by maintained OCR, reflective of the Warburg effect commonly observed in proliferating pluripotent cells.^66,67^ The preserved ECAR > OCR relationship across all conditions confirmed maintenance of the glycolytic preference essential for pluripotency, with stable levels indicative of healthy mitochondrial function and glycolytic capacity critical for supporting rapid cell proliferation and biosynthesis of essential biomolecules.^56^ Similar metabolic behavior has been reported in other synthetic hydrogel systems supporting stem cell cultures.^68,69^ Collectively, the immunostaining, flow cytometry, gene expression, and metabolic potential analyses strongly corroborate each other, confirming that the 2 : 4 : 94 P400 terpolymer provided an effective and supportive microenvironment for hPSC culture. These findings reinforce the potential of thermoresponsive synthetic scaffolds as viable, xeno-free alternatives for pluripotent stem cell cultivation and downstream applications in regenerative medicine and disease modeling.

### 3.3. Consistent pluripotency maintenance of hPSCs in 3D terpolymer cultures across sequential passages

Building upon our promising 2D culture results, we investigated the capability of the NiPAAm-*co*-4-VPBA-*co*-PEGMMA terpolymer scaffold to support multi-passage of iPSC culture in a three-dimensional environment. The transition to 3D culture is critical for advancing toward therapeutic applications, as it provides a more physiologically relevant microenvironment that better mimics native tissue architecture and cell–cell interactions compared to conventional 2D systems. This three-dimensional context is essential for scalable manufacturing processes in regenerative medicine, where maintaining pluripotency while enabling efficient cell expansion and harvest represents a central challenge for clinical translation.^44,70,71^

**Fig. 3:**
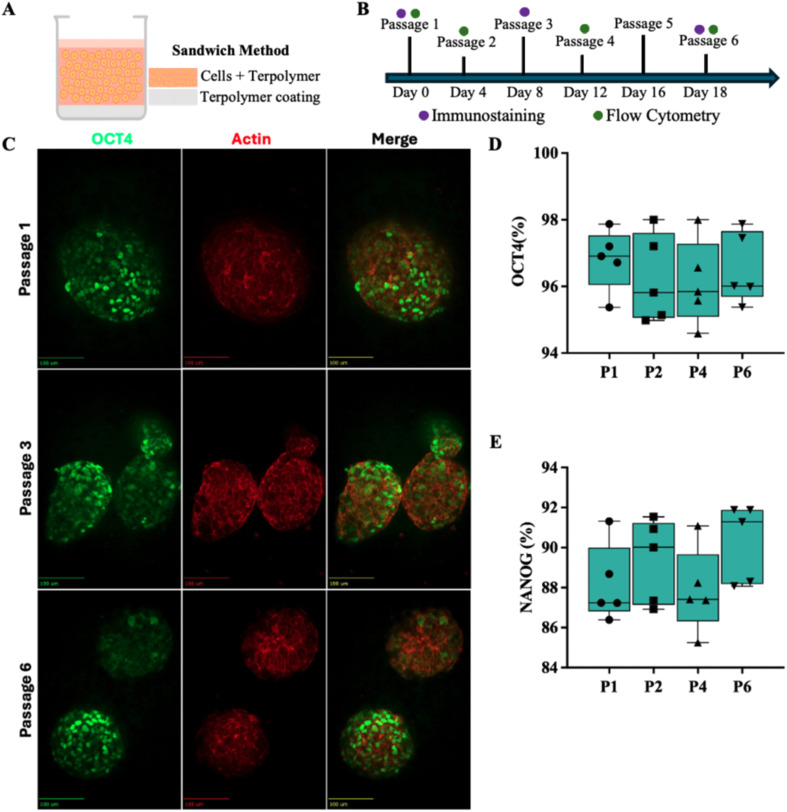
Maintenance of pluripotency in iPSCs cultured in 3D terpolymer scaffolds with RGD over multiple passages. (A) Schematic representation of the “sandwich” method used for 3D encapsulation of iPSCs in the terpolymer scaffold. (B) A comprehensive multi-passage analysis was performed to evaluate the stability of the 3D culture system supported by the terpolymer scaffold with RGD. (C) Fluorescence microscopy images showing OCT4 expression (green), actin cytoskeleton (red), and merged channels for WTC-11 iPSCs cultured in 3D terpolymer scaffolds across passages 1, 3, and 6. Scale bars represent 100 μm. (D) Flow cytometry quantification of OCT4 expression across passages 1, 2, 4, and 6 (*n* = 5). (E) Flow cytometry quantification of NANOG expression across the same passages (*n* = 5).

The terpolymer's thermoresponsive properties offered the distinct advantage of facilitating cell recovery during passaging, which helped preserve cell integrity for maintaining pluripotency of hPSCs.^49^ To create the 3D microenvironment, the sandwich culture method was employed, where a base layer of terpolymer was first applied to prevent cell attachment to the well bottom, followed by a mixture of cells with terpolymer solution (Fig. 3A). This encapsulation approach allowed for true three-dimensional cell–matrix interactions throughout the culture period, similar to strategies employed by Caiazzo *et al.*, who demonstrated successful 3D encapsulation of pluripotent stem cells in defined hydrogels.^72^

As shown in Fig. 3B, a comprehensive multi-passage analysis to evaluate the stability of the 3D culture system supported by the terpolymer scaffold with RGD was performed. Fluorescence microscopy revealed robust OCT4 expression and proper cytoskeletal organization in WTC-11 iPSCs cultured within the 3D terpolymer matrix across multiple passages (Fig. 3C). The spheroids maintained their structural integrity and pluripotent state through at least six passages, indicating stability of the culture system. The maintained expression pattern suggested that the 3D microenvironment effectively supported the preservation of pluripotency-associated transcription factors without compromising self-renewal capacity.^73,74^ Similar maintenance of pluripotency in 3D synthetic environments has been reported and validated the importance of suitable synthetic matrices for long-term stem cell culture.^64,75^

Flow cytometry analysis provided quantitative confirmation of these observations, with OCT4 expression remaining consistently above 94% (Fig. 3D) and NANOG above 85% (Fig. 3E) across all passages examined. These expression levels parallel those observed in conventional BME-based culture systems (typically 95–98% for OCT4 and 88–90% for NANOG), demonstrating the efficacy of the synthetic terpolymer in maintaining stemness even after repeated passaging cycles.^55,76,77^ Complementing these results, Fig. S1, also based on flow cytometry, demonstrated that both WTC-11 and H9 cell lines maintained their pluripotency after 6 passages when cultured with RGD-functionalized terpolymer, further validating the universal applicability of this synthetic scaffold across different pluripotent stem cell types. The consistent expression profile is particularly noteworthy, as successful long-term maintenance of pluripotency is essential for applications requiring large-scale cell expansion, including regenerative medicine and disease modeling.^78,79^

### 3.4. Biofunctionalization with ECM-derived molecules enhances hPSC expansion in 3D terpolymer environments

To further investigate the versatility of our 3D culture system, the performance of the basic terpolymer formulation (T) with variants incorporating bioactive molecules, specifically fibronectin (T + FB), RGD peptide (T + RGD), and vitronectin (T + VN), using both WTC-11 iPSCs and H9 ESCs was compared. The commercially available VitroGel™ was selected as a 3D control because it is a synthetic hydrogel,^80,81^ providing a consistent and well-validated model for benchmarking 3D culture performance. Vitronectin was incorporated since it is a key extracellular matrix protein that supports cell adhesion, survival, and proliferation, and has been shown to enhance the maintenance of pluripotency in stem cell cultures, especially in synthetic and xeno-free systems.^82,83^

**Fig. 4:**
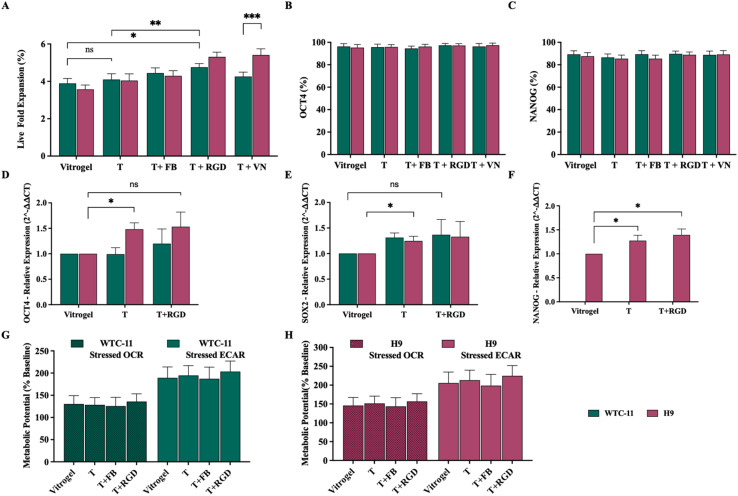
Maintenance of pluripotency and metabolic activity of hPSCs in 3D terpolymer culture. (A) Live fold expansion of iPSCs (WTC-11) and hESCs (H9) encapsulated in 2 : 4 : 94 P400 terpolymer (T) at 15 wt% compared to VitroGel™ commercial synthetic matrix control, with and without addition of fibronectin (T + FB), RGD peptide (T + RGD), or vitronectin (T + VN). (B) Flow cytometry analysis showing percentage of cells expressing pluripotency markers OCT4 and (C) NANOG, across different culture conditions. (D) Relative gene expression of OCT4 and (E) SOX2 assessed by qPCR for WTC-11 and H9 cells. (F) Relative expression of NANOG assessed by qPCR for H9 cells. (G) Metabolic potential measured by Seahorse analysis showing stressed oxygen consumption rate (OCR) and extracellular acidification rate (ECAR) for WTC-11 and (H) H9 cells, respectively. Bars represent mean ± SD (*n* = 5 for all analyses except gene expression where *n* = 3). Statistical significance: *p < 0.05, **p = 0.0123, ***p = 0.0004.

Live fold expansion analysis revealed that the base terpolymer supported cellular proliferation comparable to VitroGel™ for both cell lines (Fig. 4A). Notably, the incorporation of RGD peptide significantly enhanced proliferation for both WTC-11 and H9 cell lines, outperforming the VitroGel™ control. These findings align with two-dimension results that demonstrated RGD-containing motifs enhanced integrin-specific engagement and improve stem cell expansion. Interestingly, the addition of vitronectin showed differential effects between cell lines, with H9 cells demonstrating significantly higher proliferation compared to WTC-11 cells, which showed low improvement over the control. This cell-specific response to vitronectin likely reflects differential expression of integrin receptors between iPSCs and ESCs, consistent with reports who documented different expression of vitronectin-binding integrins (particularly αVβ5) in stem cell lines.^61,84^

Pluripotency maintenance, assessed by flow cytometry, demonstrated high expression of OCT4 (>93%) and NANOG (>87%) across all conditions for both cell lines, with no statistically significant differences respect to the VitroGel™ control (Fig. 4B and C). In addition, gene expression analysis by qPCR revealed interesting cell line-specific patterns. For OCT4 expression (Fig. 4D), H9 cells cultured in the terpolymer alone showed significantly higher expression compared to VitroGel™, while WTC-11 cells maintained comparable expression levels to the control. SOX2, which is a measure of the stem cell self-renewal capacity and a critical component of the pluripotency regulatory network, showed enhanced expression in terpolymer culture for both cell lines (Fig. 4E), though the effect was more pronounced in H9 cells compared to WTC-11 cells. Notably, NANOG expression in H9 cells (Fig. 4F) was substantially increased in both unmodified terpolymer and terpolymer with RGD compared to VitroGel™.

Metabolic profiling using Seahorse analysis demonstrated that both WTC-11 and H9 cells maintained healthy metabolic signatures in the 3D terpolymer environment, with no statistically significant differences compared to VitroGel™ control conditions. For WTC-11 cells, OCR values for terpolymer alone (128.4 ± 16.2%) and terpolymer with fibronectin (125.8 ± 19.5%) or RGD (135.9 ± 17.3%) were statistically comparable to VitroGel™ control (132.6 ± 18.9%), while ECAR values similarly showed no significant differences across conditions (T: 194.7 ± 22.1%, T + FB: 187.2 ± 26.3%, T + RGD: 203.4 ± 23.8% *vs.* VitroGel™: 189.3 ± 24.6%). H9 cells exhibited the same pattern, with OCR values showing no significant variation between terpolymer formulations (T: 151.3 ± 19.6%, T + FB: 143.7 ± 22.8%, T + RGD: 156.9 ± 20.2%) and VitroGel™ (145.8 ± 21.4%). ECAR measurements also remained statistically equivalent across all conditions (T: 213.2 ± 26.4%, T + FB: 198.5 ± 30.1%, T + RGD: 224.6 ± 27.3% *vs.* VitroGel™: 205.7 ± 28.9%). The preserved ECAR > OCR relationship across all conditions confirmed that both iPSCs and ESCs maintained their characteristic glycolytic preference essential for pluripotency.^68,85,86^ The lack of significant metabolic differences confirmed that the terpolymer formulations, with or without bioactive additives, successfully maintained the pluripotent state in 3D culture.

Overall, these findings demonstrated that the NiPAAm-*co*-4-VPBA-*co*-PEGMMA terpolymer not only supports short-term culture but also enabled maintenance of pluripotent stem cells in a 3D environment across multiple passages. The incorporation of bioactive molecules, particularly RGD peptide and vitronectin, further enhanced proliferation, while maintaining pluripotency. The differential response between iPSCs and ESCs to certain formulations highlights the importance of tailoring synthetic matrices to specific cell types and applications, as previously suggested by Amitrano *et al.*^55^ and Mulero-Russe *et al.*^64^

### 3.5. Encapsulation in terpolymer scaffolds allow hPSCs differentiation to cardiomyocytes

**Fig. 5:**
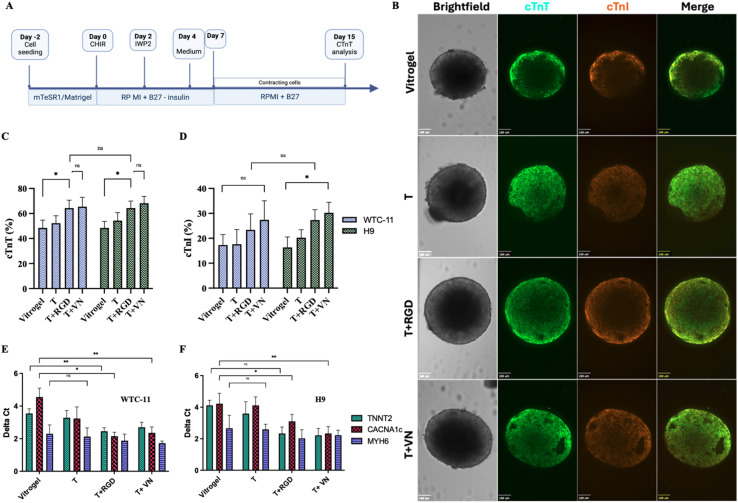
Encapsulation in terpolymer scaffolds promotes hPSC aggregation and differentiation. (A) Schematic representation of the differentiation protocol from hiPSCs/hESCs into cardiomyocytes within BME and synthetic scaffolds.^53,54^ (B) Representative confocal fluorescence images showing expression of cardiac-specific markers: cardiac Troponin T (cTnT, green), cardiac Troponin I (cTnI, orange), and merged channels in WTC-11 iPSC-derived cardiomyocytes encapsulated in the terpolymer scaffold. Scale bars: 100 μm. (C) Flow cytometry quantification of cTnT and (D) cTnI expression for WTC-11 and H9-derived cardiomyocytes after 15 days of differentiation compared to VitroGel™ (*n* = 5). (E) Relative gene expression analysis by qPCR of cardiac differentiation markers TNNT2, CACNA1c, and MYH6 for (E) WTC-11 and (F) H9 cell lines cultured in 3D terpolymer scaffolds (*n* = 3). Data shown as mean ± SD. Statistical significance: *p < 0.05, **p < 0.001.

After establishing that hPSCs could be effectively maintained in an undifferentiated state within 3D terpolymer environments, we proceeded to investigate their capacity for directed differentiation into cardiomyocytes. An optimized protocol, illustrated in Fig. 5A, which included temporal modulation of Wnt signaling through CHIR99021 and IWP2 treatments, was used to induce cardiac lineage differentiation.^53,54^ When encapsulated within the 3D terpolymer environment, both WTC-11 and H9 cells demonstrated differentiation after 15 days of differentiation. Confocal microscopy analysis revealed the expression of cardiac-specific markers within the differentiated cells (Fig. 5B). Cells exhibited strong immunoreactivity for cardiac troponin T (cTnT), a critical component of the sarcomeric thin filament that regulates muscle contraction, as well as cardiac troponin I (cTnI), which serves as a key indicator of cardiomyocyte maturation.^53,55^

Flow cytometry analysis was utilized to quantify the expression of cardiac-specific proteins, enabling precise determination of differentiation efficiency and cardiomyocyte maturation. Quantitative assessment *via* flow cytometry confirmed these observations, revealing that approximately 60% of cells expressed cTnT when differentiated within the unmodified terpolymer scaffold (Fig. 5C). This efficiency was comparable to that observed in the VitroGel™ control, indicating that the synthetic terpolymer provided an equally supportive microenvironment for cardiac differentiation. Notably, the modification of the terpolymer with bioactive molecules significantly enhanced differentiation outcomes. The incorporation of RGD peptide and vitronectin resulted in increased cTnT expression (65.8 ± 5.2% and 66.2 ± 5.9%, respectively) compared to the unmodified terpolymer (49.7 ± 5.8%). This efficiency is comparable to previously reported differentiation efficiencies using BME-based protocols, which typically yield between 50–70% cTnT-positive cells.^41,42,87^ Similarly, the expression of cTnI, showed a parallel trend (Fig. 5D). While the unmodified terpolymer supported cTnI expression at levels comparable to VitroGel™ (approximately 17%), the addition of RGD peptide and vitronectin resulted in significantly higher expression (23.5 ± 6.8% and 26.8 ± 7.1%, respectively). The enhanced cTnI expression with bioactive modifications suggests improved cardiomyocyte maturation, as cTnI is a late-stage cardiac marker that indicates functional contractile apparatus development. This improvement demonstrated the importance of integrin–ECM interactions in driving cardiac lineage progression beyond initial specification.^8^ The improvements in both cTnT and cTnI expression likely stem from enhanced integrin-mediated signaling, as demonstrated by Santoro *et al.* and Israeli-Rosenberg *et al.*, suggesting that the bioactive-modified scaffolds not only improved differentiation efficiency, but also promoted cardiomyocyte maturation, addressing a key challenge in cardiac tissue engineering.^88–90^

Gene expression analysis *via* quantitative PCR was performed to evaluate transcriptional changes associated with cardiac lineage commitment and functional maturation. Gene expression analysis *via* qPCR provided further evidence of successful cardiac differentiation within the terpolymer scaffolds (Fig. 5E and F). For both WTC-11 and H9 cell lines, a substantial upregulation of key cardiac genes was observed, including TNNT2 (encoding cardiac troponin T), CACNA1c (encoding the alpha subunit of the L-type calcium channel essential for cardiac excitation-contraction coupling), and MYH6 (encoding alpha-myosin heavy chain, a primary contractile protein in the heart).^53^ Together, these molecular changes represented the fundamental transition from pluripotent stem cells to functional cardiac cells. The expression levels of these genes were comparable between the terpolymer and VitroGel™ conditions, with slight enhancements observed in the bioactive-modified scaffolds, particularly for CACNA1c in the terpolymer with vitronectin condition. These expression patterns indicated cardiac lineage and the acquisition of key cardiomyocytes characteristic within our system, similar to observations by Vučković *et al.*, who characterized the metabolic maturation in iPSC-derived cardiomyocytes.^67^

The metabolic profile of the differentiated cardiomyocytes within the terpolymer scaffolds provided additional insights into their functional maturation. Seahorse analysis revealed that the iPSCs maintained healthy metabolic, with OCR and ECAR comparable to those observed in the VitroGel™ control (Fig. S2). Notably, there were no significant differences in metabolic potential across the different culture conditions, indicating that the terpolymer environment, with or without bioactive modifications, adequately supported the metabolic requirements of differentiating cardiomyocytes. This finding is particularly important as the metabolic shift from glycolysis to oxidative phosphorylation is a key feature of cardiomyocyte maturation, as demonstrated by Correia *et al.*, who showed that 3D aggregate culture improved metabolic maturation of human pluripotent stem cell-derived cardiomyocytes.^66^ Additional characterization, including cTnT expression *via* flow cytometry and confirmation by immunostaining of cardiomyocytes differentiated in 2D conditions is presented in Fig. S3, allowing direct comparison between 2D and 3D differentiation efficiency.

### 3.6. Tunable mechanical properties through terpolymer blending enables precise tailoring of scaffold stiffness

Building upon previous work characterizing individual NiPAAm-*co*-4-VPBA-*co*-PEGMMA terpolymers where LCST, stiffness, and other critical properties were assessed, the investigation intended to explore how blending these polymers could further enhance their versatility for diverse cell culture applications requiring specific mechanical properties.^49–51^ An earlier publication demonstrated that varying the molar ratios of NiPAAm, VPBA, and PEGMMA monomers, along with the molecular weight of PEGMMA (P400 or P1000), yielded terpolymers with a range of thermal and mechanical characteristics. This foundational work established individual terpolymers with specific properties; however, creating a wider spectrum of scaffold characteristics while maintaining physiologically relevant thermal behavior remained challenging.^91^

The current study addressees this limitation by systematically blending selected terpolymers to create matrices with intermediate properties. Terpolymer blend combinations were analyzed to establish their potential for creating customized cellular microenvironments. As an example, when blending 2 : 3 : 95 P400 with 10 : 4 : 86 P1000, it was observed that increasing the proportion of 10 : 4 : 86 P1000 progressively reduced scaffold stiffness from 19.1 kPa to 6.4 kPa, while shifting the LCST from 34.5 °C toward 43.1 °C (Fig. S4A). Similarly, the combination of 2 : 3 : 95 P400 with 4 : 8 : 88 P400 demonstrated that higher proportions of 4 : 8 : 88 P400 decreased stiffness, while elevating the LCST (Fig. S4B).

**Fig. 6:**
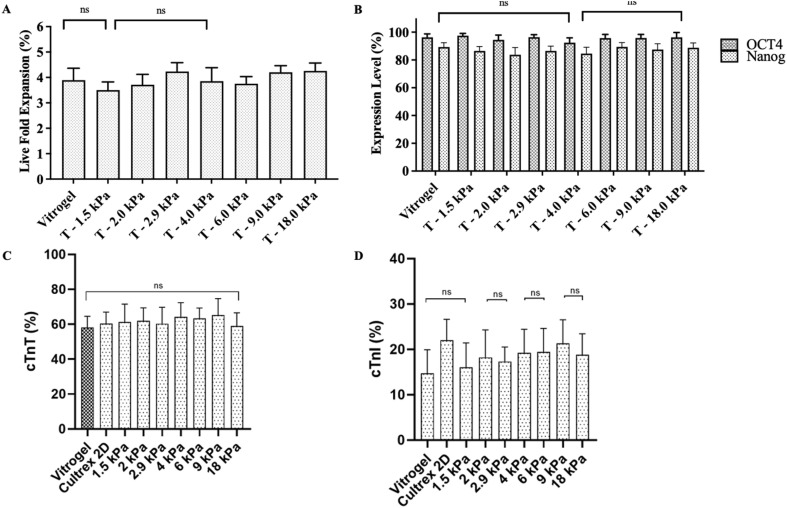
Effect of scaffold stiffness on iPSC proliferation and differentiation into cardiomyocytes. (A) Live fold expansion rates of WTC-11 iPSCs cultured on terpolymer scaffolds with varying stiffness values and control substrates. (B) Expression of pluripotency markers OCT4 and NANOG in iPSCs across different terpolymer stiffness conditions. (C) Percentage of cells expressing cTnT following differentiation on various terpolymer formulations. (D) Percentage of cells expressing cTnI across identical culture conditions. Statistical significance indicated as: ns = not significant. Data represents mean ± SD, *n* = 4.

As a result, several blend formulations that maintained an LCST close to physiological temperature (37 °C), were targeted since they are particularly suitable for cell culture applications (Table S2). For instance, a 90 : 10 blend of 2 : 3 : 95 P400 and 10 : 4 : 86 P1000 produced a scaffold with 17.7 kPa stiffness and an LCST of 36.5 °C, while a 70 : 30 mixture of 4 : 4 : 92 P400 and 4 : 12 : 84 P1000 yielded a softer matrix (7.4 kPa) with an LCST of 37.6 °C. The relationship observed between blend composition and resulting properties enables predictable scaffold engineering for specific applications. These near-physiological LCST blends were selected for cell culture experiments, allowing the examination of how scaffold rigidity influenced cell behavior, while maintaining consistent thermal responsiveness. When assessed with iPSCs, terpolymer blends with stiffness values ranging from 1.5 to 18.0 kPa and functionalized with RGD peptide, all supported comparable cell proliferation rates (Fig. 6A). Expression of pluripotency markers OCT4 and Nanog remained consistently high (>95% and >87%, respectively) across all stiffness conditions (Fig. 6B), indicating that iPSC pluripotency was robustly maintained within this mechanical range.

This finding aligns with several recent studies that have also reported mechanical insensitivity within specific ranges or conditions. For example, Eroshenko *et al.* demonstrated that early differentiation of human embryonic stem cells appears to follow a stochastic process across different PDMS substrate stiffnesses, with some cells maintaining pluripotency markers regardless of mechanical cues.^92^ Similarly, Llewellyn *et al.* showed that while substrate stiffness can modulate hiPSC differentiation competence, different hPSC lines could react differentially to substrate stiffness, suggesting that additional unknown factors present in culture conditions might cooperate with substrate mechanical properties.^93^ Cozzolino *et al.* found that liver stem cells showed context-dependent responses to substrate stiffness, with some cellular processes being more influenced by biochemical rather than mechanical factors within certain ranges.^94^ These studies collectively demonstrate that stem cell response to substrate stiffness can be context-dependent, with some cell types or culture conditions showing minimal sensitivity to mechanical cues within certain ranges. However, this contrasts with the well-established paradigm from studies by Paiva *et al.* and others showing clear stiffness-dependent lineage specification.^95^ The apparent mechanical insensitivity of pluripotency maintenance observed in our system suggests that other factors, including the presence of bioactive molecules like RGD peptide and the three-dimensional nature of the encapsulation, may play a more dominant role in regulating iPSC behavior than substrate stiffness alone within the tested range.

For cardiac differentiation applications, a series of tailored terpolymer blends spanning 2.2–9.0 kPa (Table 1) were created. All formulations supported comparable levels of cTnT (∼60%), suggesting that differentiation efficiency was not significantly affected by substrate rigidity within this range (Fig. 6C). The cTnI expression levels also remained consistent across the different stiffness conditions (Fig. 6D), indicating that matrix rigidity within this range did not significantly impact cardiomyocyte maturation markers. However, cells cultured on the 9.0 kPa scaffold exhibited improved contractile behavior, which aligns with the mechanical properties of native myocardium.^96,97^ This observation suggested that while expression of cardiac markers may not be significantly affected by substrate stiffness in this range, functional properties such as contractility may still be influenced by the mechanical microenvironment.

**Table 1:** Concentration and stiffness of terpolymers blends used as scaffold to differentiate iPSCs into cardiomyocytes

Combination (terpolymer 1–terpolymer 2)	Concentration (wt%)	Stiffness (kPa)
2 : 4 : 94 P400–2 : 4 : 94 P1000	60–40	2.2 ± 0.3
2 : 4 : 94 P400–4 : 8 : 88 P400	60–40	4.4 ± 0.2
2 : 4 : 94 P400–2 : 4 : 94 P1000	70–30	6.3 ± 0.2
2 : 4 : 94 P400–4 : 8 : 88 P400	70–30	9.0 ± 0.3

The significance of substrate mechanics for cardiac cell function has been well-documented. Previous investigations have shown that matrices mimicking the stiffness of the developing myocardium (∼10 kPa) can promote optimal sarcomere organization and contractile activity.^96,97^ Additionally, dynamic alterations in substrate rigidity during culture periods have been reported to enhance maturation by replicating the physiological stiffening that occurs during cardiac development.^41,98^ This work extends these concepts to iPSC-derived cardiomyocytes in 3D culture, demonstrating that functional cardiac properties can be modulated through mechanical cues despite minimal changes in protein marker expression.

These findings demonstrated that terpolymer blending provided a straightforward approach to generate a spectrum of scaffold properties, while maintaining physiologically relevant thermal characteristics. The ability to create scaffolds with precise LCST values near 37 °C and independently controllable mechanical stiffness significantly expands the utility of these synthetic matrices for applications requiring specific microenvironments, such as stem cell expansion and directed differentiation.

## 4. Conclusion

This study demonstrated the successful maintenance of pluripotency in both iPSCs and ESCs using a fully synthetic, xeno-free, and thermoresponsive terpolymer scaffold. The 2 : 4 : 94 P400 formulation exhibited optimal performance, enabling robust expansion, while maintaining high expression of pluripotency markers comparable to traditional biological matrices. A key advantage of this system is the precise control over mechanical properties, with scaffolds demonstrating tunable stiffness ranging from 0.5 to 18.0 kPa, covering the physiologically relevant range for various tissue types, including cardiac tissue (10–15 kPa). Compared to tunable hydrogels that typically cannot independently control mechanical properties and other functionalities, this terpolymer system uniquely combines independent control of stiffness and thermal responsiveness with thermoresponsive cell recovery capabilities, batch consistency, and dual 2D/3D compatibility in a single platform. This tunability could allow researchers to tailor the microenvironment to specific cellular requirements, addressing a significant limitation of natural matrices where mechanical properties cannot be readily adjusted.

The terpolymer scaffold supported efficient differentiation into cardiomyocytes, achieving approximately 65% cTnT-positive and 25% cTnI-positive cells. The mechanical properties of the scaffold likely played a role in this efficient differentiation, as optimal substrate stiffness has been shown to influence lineage-specific differentiation of stem cells. Unlike conventional matrices such as Matrigel™ and Cultrex™, which exhibit inherent batch-to-batch variability due to their biological origin,^18,20^ this platform eliminates such variability and undefined composition, offering reproducibility and scalability essential for clinical translation. The incorporation of bioactive motifs like RGD peptides and vitronectin further optimized cellular expansion, aggregation, and differentiation processes. Additionally, the scaffold's thermoresponsive nature enabled non-invasive, gentle cell recovery, preserving cellular integrity for downstream applications. The consistent metabolic activity observed across conditions confirmed the ability of the scaffold to support the energetic demands of both pluripotent and differentiated cells.

Collectively, these results position this tunable terpolymer system as a potential versatile tool for stem cell research, regenerative medicine, disease modeling, and drug discovery. Its mechanical tunability, reproducibility, and compatibility with both 2D and 3D culture platforms make it a significant advancement toward engineering functional tissues and developing next-generation therapeutic strategies for cardiac and other diseases. Future research should explore the use of this thermoresponsive terpolymer scaffold for the differentiation of hPSCs into other specialized cell types, such as hepatocytes, neurons, intestinal organoids and pancreatic beta cells, potentially with tailored stiffness parameters optimized for each specific lineage.

## Conflicts of interest

This work is the subject of intellectual property filings by the University of Puerto Rico, including a pending patent titled “Synthetic Matrixes for Cell Culture and Manufacture” (PCT/US2021/061256) and an invention disclosure titled “Synthetic Polymer Blends with Broad Mechanical Properties for Cell Culture” (25-007-DISC-UPR).

## Supplementary Material

RA-015-D5RA04674B-s001

## Data Availability

Data supporting this article have been included as part of the SI. See DOI: https://doi.org/10.1039/d5ra04674b.

## References

[cit1] Peischard S., Piccini I., Strutz-Seebohm N., Greber B., Seebohm G. (2017). From iPSC towards cardiac tissue—a road under construction. Pflugers Arch..

[cit2] Macqueen L. A. (2018). *et al.*
, A tissue-engineered scale model of the heart ventricle. Nat. Biomed. Eng..

[cit3] Depalma S. J., Davidson C. D., Stis A. E., Helms A. S., Baker B. M. (2021). Microenvironmental determinants of organized iPSC-cardiomyocyte tissues on synthetic fibrous matrices. Biomater. Sci..

[cit4] Le M. N. T., Hasegawa K. (2019). Expansion culture of human pluripotent stem cells and production of cardiomyocytes. Bioengineering.

[cit5] Keung A. J., Dong M., Schaffer D. V., Kumar S. (2013). Pan-neuronal maturation but not neuronal subtype differentiation of adult neural stem cells is mechanosensitive. Sci. Rep..

[cit6] Tomaskovic-Crook E., Gu Q., Rahim S. N. A., Wallace G. G., Crook J. M. (2020). Conducting Polymer Mediated Electrical Stimulation Induces Multilineage Differentiation with Robust Neuronal Fate Determination of Human Induced Pluripotent Stem Cells. Cells.

[cit7] Li R. K. and Weisel R. D.,Cardiac Regeneration and Repair: Pathology and Therapies, 2014, vol. 1

[cit8] Schwach V., Passier R. (2019). Native cardiac environment and its impact on engineering cardiac tissue. Biomater. Sci..

[cit9] Ho T. C. (2022). *et al.*
, Hydrogels: Properties and Applications in Biomedicine. Molecules.

[cit10] Fontaine V. (2021). *et al.*
, Generation of iPSC line from MYH7 R403L mutation carrier with severe hypertrophic cardiomyopathy and isogenic CRISPR/Cas9 corrected control. Stem Cell Res..

[cit11] Jacot J. G., Martin J. C., Hunt D. L. (2010). Mechanobiology of cardiomyocyte development. J. Biomech..

[cit12] Li Y. (2017). *et al.*
, Engineering-derived approaches for iPSC preparation, expansion, differentiation and applications. Biofabrication.

[cit13] Rikhtegar R. (2019). *et al.*
, Stem cells as therapy for heart disease: iPSCs, ESCs, CSCs, and skeletal myoblasts. Biomed. Pharmacother..

[cit14] Arrieta-Viana L. F., García A. J. (2025). Spatiotemporally-patterned biomaterials for organoid culture. Curr. Opin. Biomed. Eng..

[cit15] Barreto-Gamarra C., Domenech M. (2017). Integrin stimulation by collagen I at the progenitor stage accelerates maturation of human iPSC-derived cardiomyocytes. J. Mol. Cell. Cardiol..

[cit16] Brooks E. A., Gencoglu M. F., Corbett D. C., Stevens K. R., Peyton S. R. (2019). An omentum-inspired 3D PEG hydrogel for identifying ECM-drivers of drug resistant ovarian cancer. APL Bioeng..

[cit17] Wang H., Luo X., Leighton J. (2015). Extracellular Matrix and Integrins in Embryonic Stem Cell Differentiation. Biochem. Insights.

[cit18] Hughes C. S., Postovit L. M., Lajoie G. A. (2010). Matrigel: a complex protein mixture required for optimal growth of cell culture. Proteomics.

[cit19] Ferreira L. A. , do Nascimento D. F., Tandon I., Cordes L. and Balachandran K., The future is fully defined: recombinant fragment E8 of laminin-511 is a viable xenofree alternative to Matrigel for hiPSC culture and differentiation into neurovascular cell types, Biorxiv, Preprint 10.1101/2024.06.20.599891, 2024

[cit20] Kozlowski M. T., Crook C. J., Ku H. T. (2021). Towards organoid culture without Matrigel. Commun. Biol..

[cit21] Nagy A. and Turksen K., Induced Pluripotent Stem (IPS) Cells Methods and Protocols, Springer Protocols, 2nd edn, 2022

[cit22] Wang L., Hu D., Xu J., Hu J., Wang Y. (2024). Complex in vitro model: A transformative model in drug development and precision medicine. Clin. Transl.
Sci..

[cit23] Okamoto M., John B. (2013). Synthetic biopolymer nanocomposites for tissue engineering scaffolds. Prog. Polym. Sci..

[cit24] Janoušková O. (2018). Synthetic polymer scaffolds for soft tissue engineering. Physiol. Res..

[cit25] Villa-Diaz L. G. (2010). *et al.*
, Synthetic polymer coatings for long-term growth of human embryonic stem cells. Nat. Biotechnol..

[cit26] Kawase E., Nakatsuji N. (2023). Development of substrates for the culture of human pluripotent stem cells. Biomater. Sci..

[cit27] Shimizu E. (2022). *et al.*
, A chemically-defined plastic scaffold for the xeno-free production of human pluripotent stem cells. Sci. Rep..

[cit28] Timilsina S., McCandliss K. F., Trivedi E., Villa-Diaz L. G. (2023). Enhanced Expansion of Human Pluripotent Stem Cells and Somatic Cell Reprogramming Using Defined and Xeno-Free Culture Conditions. Bioengineering.

[cit29] Yi B., Xu Q., Liu W. (2022). An overview of substrate stiffness guided cellular response and its applications in tissue regeneration. Bioact. Mater..

[cit30] Xue B., Tang D., Wu X., Xu Z., Gu J., Han Y., Zhu Z., Qin M., Zou X., Wang W., Cao Y. (2021). Engineering hydrogels with homogeneous mechanical properties for controlling stem cell lineage specification. Proc. Natl. Acad. Sci. U.S.A..

[cit31] Sacchetto C., Vitiello L., de Windt L. J., Rampazzo A., Calore M. (2020). Modeling cardiovascular diseases with hipsc-derived cardiomyocytes in 2d and 3d cultures. Int. J. Mol. Sci..

[cit32] Ravi M., Paramesh V., Kaviya S. R., Anuradha E., Paul Solomon F. D. (2015). 3D cell culture systems: Advantages and applications. J. Cell. Physiol..

[cit33] Pérez Del Río E., Martinez Miguel M., Veciana J., Ratera I., Guasch J. (2018). Artificial 3D Culture Systems for T Cell Expansion. ACS Omega.

[cit34] Murphy A. R., Laslett A., O’Brien C. M., Cameron N. R. (2017). Scaffolds for 3D in vitro culture of neural lineage cells. Acta Biomater..

[cit35] Ekerdt B. L. (2018). *et al.*
, Thermoreversible Hyaluronic Acid-PNIPAAm Hydrogel Systems for 3D Stem Cell Culture. Adv. Healthcare Mater..

[cit36] Schussler O., Chachques J. C., Alifano M., Lecarpentier Y. (2022). Key Roles of RGD-Recognizing Integrins During Cardiac Development, on Cardiac Cells, and After Myocardial Infarction. J. Cardiovasc. Transl. Res..

[cit37] Zhou P. (2018). *et al.*
, Molecular basis for RGD-containing peptides supporting adhesion and self-renewal of human pluripotent stem cells on synthetic surface. Colloids Surf., B.

[cit38] Prowse A. B. J. (2010). *et al.*
, Long term culture of human embryonic stem cells on recombinant vitronectin in ascorbate free media. Biomaterials.

[cit39] Heydarkhan-Hagvall S. (2012). *et al.*
, The effect of vitronectin on the differentiation of embryonic stem cells in a 3D culture system. Biomaterials.

[cit40] Martino M. M. (2009). *et al.*
, Controlling integrin specificity and stem cell differentiation in 2D and 3D environments through regulation of fibronectin domain stability. Biomaterials.

[cit41] Zhao M., Tang Y., Zhou Y., Zhang J. (2019). Deciphering Role of Wnt Signalling in Cardiac Mesoderm and Cardiomyocyte Differentiation from Human iPSCs: Four-dimensional control of Wnt pathway for hiPSC-CMs differentiation. Sci. Rep..

[cit42] Melkoumian Z. (2010). *et al.*
, Synthetic peptide-acrylate surfaces for long-term self-renewal and cardiomyocyte differentiation of human embryonic stem cells. Nat. Biotechnol..

[cit43] Ovadia E. M., Colby D. W., Kloxin A. M. (2018). Designing well-defined photopolymerized synthetic matrices for three-dimensional culture and differentiation of induced pluripotent stem cells. Biomater. Sci..

[cit44] Villa-Diaz L. G., Kim J. K., Lahann J., Krebsbach P. H. (2014). Derivation and Long-Term Culture of Transgene-Free Human Induced Pluripotent Stem Cells on Synthetic Substrates. Stem Cells Transl. Med..

[cit45] Grijalva Garces D., Radtke C. P., Hubbuch J. (2022). A Novel Approach for the Manufacturing of Gelatin-Methacryloyl. Polymers.

[cit46] Arguchinskaya N. V. (2023). *et al.*
, Properties and Printability of the Synthesized Hydrogel Based on GelMA. Int. J. Mol. Sci..

[cit47] Wang Q. (2022). *et al.*
, Circular Patterns of Dynamic Covalent Hydrogels with Gradient Stiffness for Screening of the Stem Cell Microenvironment. ACS Appl. Mater. Interfaces.

[cit48] Ueda N., Sawada S., Yuasa F., Kato K., Nagahama K. (2022). Covalent Stem Cell-Combining Injectable Materials with Enhanced Stemness and Controlled Differentiation in Vivo. ACS Appl. Mater. Interfaces.

[cit49] Lizana-Vasquez G. D., Arrieta-Viana L. F., Mendez-Vega J., Acevedo A., Torres-Lugo M. (2022). Synthetic Thermo-Responsive Terpolymers as Tunable Scaffolds for Cell Culture Applications. Polymers.

[cit50] Lizana-Vasquez G. D., Mendez-Vega J., Cappabianca D., Saha K., Torres-Lugo M. (2024). In vitro encapsulation and expansion of T and CAR-T cells using 3D synthetic thermo-responsive matrices. RSC Adv..

[cit51] Lizana-Vasquez G. D. (2024). *et al.*
, In Vitro Assessment of Thermo-Responsive Scaffold as a 3D Synthetic Matrix for CAR-T Potency Testing Against Glioblastoma Spheroids. J Biomed Mater Res A.

[cit52] Ludwig T. E. (2006). *et al.*
, Derivation of human embryonic stem cells in defined conditions. Nat. Biotechnol..

[cit53] Lian X. (2012). *et al.*
, Robust cardiomyocyte differentiation from human pluripotent stem cells via temporal modulation of canonical Wnt signaling. Proc. Natl. Acad. Sci. U. S. A..

[cit54] Lian X. (2013). *et al.*
, Directed cardiomyocyte differentiation from human pluripotent stem cells by modulating Wnt/β-catenin signaling under fully defined conditions. Nat. Protoc..

[cit55] Amitrano M. J., Cho M., Coughlin E. M., Palecek S. P., Murphy W. L. (2025). Synthetic hydrogels support robust and reproducible cardiomyocyte differentiation. Biomater. Sci..

[cit56] Fasano C., Cavaliere A., Tiranti V., Peron C. (2024). Protocol for evaluating mitochondrial respiration in iPSC-derived neurons by the Seahorse XF analyzer. STAR Protoc..

[cit57] Stempien A. (2024). *et al.*
, Influence of Remodeled ECM and Co-culture with iPSC-Derived Cardiac Fibroblasts on the Mechanical Function of Micropatterned iPSC-Derived Cardiomyocytes. Cardiovasc. Eng. Technol..

[cit58] Lou Y. R. (2015). *et al.*
, Silica bioreplication preserves three-dimensional spheroid structures of human pluripotent stem cells and HepG2 cells. Sci. Rep..

[cit59] Maldonado M. (2015). *et al.*
, The effects of electrospun substrate-mediated cell colony morphology on the self-renewal of human induced pluripotent stem cells. Biomaterials.

[cit60] Brafman D. A. (2010). *et al.*
, Long-term human pluripotent stem cell self-renewal on synthetic polymer surfaces. Biomaterials.

[cit61] Brafman D. A., Phung C., Kumar N., Willert K. (2013). Regulation of endodermal differentiation of human embryonic stem cells through integrin-ECM interactions. Cell Death Differ..

[cit62] Burgess K. A. (2021). *et al.*
, Functionalised peptide hydrogel for the delivery of cardiac progenitor cells. Mater. Sci. Eng., C.

[cit63] Wu S., Wang H., Ren Y., Liu Y., Wen X. (2025). Generation of induced pluripotent stem cell-derived anterior foregut endoderms on integrin-binding short peptide-based synthetic substrates. Biomed. Mater..

[cit64] Mulero-Russe A. (2025). *et al.*
, Synthetic hydrogel substrate for human induced pluripotent stem cell definitive endoderm differentiation. Biomaterials.

[cit65] Nakashima Y., Tsukahara M. (2022). Laminin-511 Activates the Human Induced Pluripotent Stem Cell Survival via α6β1 Integrin-Fyn-RhoA-ROCK Signaling. Stem Cells Dev..

[cit66] Correia C. (2018). *et al.*
, 3D aggregate culture improves metabolic maturation of human pluripotent stem cell derived cardiomyocytes. Biotechnol. Bioeng..

[cit67] Vučković S. (2022). *et al.*
, Characterization of cardiac metabolism in iPSC-derived cardiomyocytes: lessons from maturation and disease modeling. Stem Cell Res. Ther..

[cit68] Miguel V. (2023). *et al.*
, Protocol to analyze bioenergetics in single human induced-pluripotent-stem-cell-derived kidney organoids using Seahorse XF96. STAR Protoc..

[cit69] Ishida T., Nakao S., Ueyama T., Harada Y., Kawamura T. (2020). Metabolic remodeling during somatic cell reprogramming to induced pluripotent stem cells: Involvement of hypoxia-inducible factor 1. Inflammation Regener..

[cit70] Yang J. J., Liu J. F., Kurokawa T., Kitada K., Gong J. P. (2015). Hydrogels as feeder-free scaffolds for long-term self-renewal of mouse induced pluripotent stem cells. J. Tissue Eng. Regener. Med..

[cit71] Dzhoyashvili N. A., Shen S., Rochev Y. A. (2015). Natural and Synthetic Materials for Self-Renewal, Long-Term Maintenance, and Differentiation of Induced Pluripotent Stem Cells. Adv. Healthcare Mater..

[cit72] Caiazzo M. , Tabata Y. and Lutolf M., Generation of induced pluripotent stem cells in defined three-dimensional hydrogels, in Methods in Molecular Biology, Humana Press Inc., 2017, vol. 1612, pp. 65–78 10.1007/978-1-4939-7021-6_5 28634935

[cit73] Chhabra A. (2017). Derivation of Human Induced Pluripotent Stem Cell (iPSC) Lines and Mechanism of Pluripotency: Historical Perspective and Recent Advances. Stem Cell Rev. Rep..

[cit74] Lambshead J. W. (2018). *et al.*
, Long-Term Maintenance of Human Pluripotent Stem Cells on cRGDfK-Presenting Synthetic Surfaces. Sci. Rep..

[cit75] Loo Y. (2019). *et al.*
, A Chemically Well-Defined, Self-Assembling 3D Substrate for Long-Term Culture of Human Pluripotent Stem Cells. ACS Appl. Bio Mater..

[cit76] Zhang C. (2025). *et al.*
, Generation of an induced pluripotent stem cell (iPSC) line (INNDSUi0010-A) from a patient with Anti-MDA5 antibody-positive dermatomyositis. Stem Cell Res..

[cit77] Jiang Y. (2025). *et al.*
, Generation and Characterization of a Human-Derived iPSC line from a female child with First-Episode of sporadic schizophrenia. Stem Cell Res..

[cit78] Patel L., Worch J. C., Dove A. P., Gehmlich K. (2023). The Utilisation of Hydrogels for iPSC-Cardiomyocyte Research. Int. J. Mol. Sci..

[cit79] Nachlas A. L. Y. (2018). *et al.*
, Human iPSC-derived mesenchymal stem cells encapsulated in PEGDA hydrogels mature into valve interstitial-like cells. Acta Biomater..

[cit80] Kim M. J. (2023). *et al.*
, Reciprocal enhancement of SARS-CoV-2 and influenza virus replication in human pluripotent stem cell-derived lung organoids1. Emerging Microbes Infect..

[cit81] Pineiro-Llanes J., da Silva L., Huang J., Cristofoletti R. (2024). Comparative Study of Basement-membrane Matrices for Human Stem Cell Maintenance and Intestinal Organoid Generation. J. Visualized Exp..

[cit82] Nagaoka M., Kobayashi M., Kawai C., Mallanna S. K., Duncan S. A. (2015). Design of a vitronectin-based recombinant protein as a defined substrate for differentiation of human pluripotent stem cells into hepatocyte-like cells. PLoS One.

[cit83] Liu Y. C. (2021). *et al.*
, Laminin-511 and recombinant vitronectin supplementation enables human pluripotent stem cell culture and differentiation on conventional tissue culture polystyrene surfaces in xeno-free conditions. J. Mater. Chem. B.

[cit84] Wong J. C. Y. (2010). *et al.*
, Definitive endoderm derived from human embryonic stem cells highly express the integrin receptors αV and β5. Cell Adhes. Migr..

[cit85] Bilic J., Izpisua Belmonte J. C. (2012). Concise review: Induced pluripotent stem cells versus embryonic stem cells: Close enough or yet too far apart?. Stem Cells.

[cit86] Song J., Saha S., Gokulrangan G., Tesar P. J., Ewing R. M. (2012). DNA and chromatin modification networks distinguish stem cell pluripotent ground states. Mol. Cell. Proteomics.

[cit87] Samaras J. J., Abecasis B., Serra M., Ducci A., Micheletti M. (2018). Impact of hydrodynamics on iPSC-derived cardiomyocyte differentiation processes. J. Biotechnol..

[cit88] Tang Y. (2016). *et al.*
, Induction and differentiation of human induced pluripotent stem cells into functional cardiomyocytes on a compartmented monolayer of gelatin nanofibers. Nanoscale.

[cit89] Santoro R., Perrucci G. L., Gowran A., Pompilio G. (2019). Unchain My Heart: Integrins at the Basis of iPSC Cardiomyocyte Differentiation. Stem Cells Int..

[cit90] Israeli-Rosenberg S., Manso A. M., Okada H., Ross R. S. (2014). Integrins and integrin-associated proteins in the cardiac myocyte. Circ. Res..

[cit91] Korpanty J., Parent L. R., Hampu N., Weigand S., Gianneschi N. C. (2021). Thermoresponsive polymer assemblies via variable temperature liquid-phase transmission electron microscopy and small angle X-ray scattering. Nat. Commun..

[cit92] Eroshenko N., Ramachandran R., Yadavalli V. K., Rao R. R. (2013). Effect of substrate stiffness on early human embryonic stem cell differentiation. J. Biol. Eng..

[cit93] Llewellyn J., Charrier A., Cuciniello R., Helfer E., Dono R. (2024). Substrate stiffness alters layer architecture and biophysics of human induced pluripotent stem cells to modulate their differentiation potential. iScience.

[cit94] Cozzolino A. M. (2016). *et al.*
, Modulating the Substrate Stiffness to Manipulate Differentiation of Resident Liver Stem Cells and to Improve the Differentiation State of Hepatocytes. Stem Cells Int..

[cit95] Paiva S. (2020). *et al.*
, Polyacrylamide Hydrogels with Rigidity-Independent Surface Chemistry Show Limited Long-Term Maintenance of Pluripotency of Human Induced Pluripotent Stem Cells on Soft Substrates. ACS Biomater. Sci. Eng..

[cit96] Guimarães C. F., Gasperini L., Marques A. P., Reis R. L. (2020). The stiffness of living tissues and its implications for tissue engineering. Nat. Rev. Mater..

[cit97] Corbin E. A. (2019). *et al.*
, Tunable and Reversible Substrate Stiffness Reveals a Dynamic Mechanosensitivity of Cardiomyocytes. ACS Appl. Mater. Interfaces.

[cit98] Sahara M. (2023). Recent Advances in Generation of In Vitro Cardiac Organoids. Int. J. Mol. Sci..

