# Supplementary material for: Synthetic thermoresponsive scaffolds for the expansion and differentiation of human pluripotent stem cells into cardiomyocytes
Source: RSC Adv. 2025 Sep 2;15(38):31296–312. doi: 10.1039/d5ra04674b (PMC12402885; doi:10.1039/d5ra04674b)
Supplement: RA-015-D5RA04674B-s001 [file RA-015-D5RA04674B-s001.pdf]

Supplementary Information (SI) for RSC Advances.
This journal is © The Royal Society of Chemistry 2025

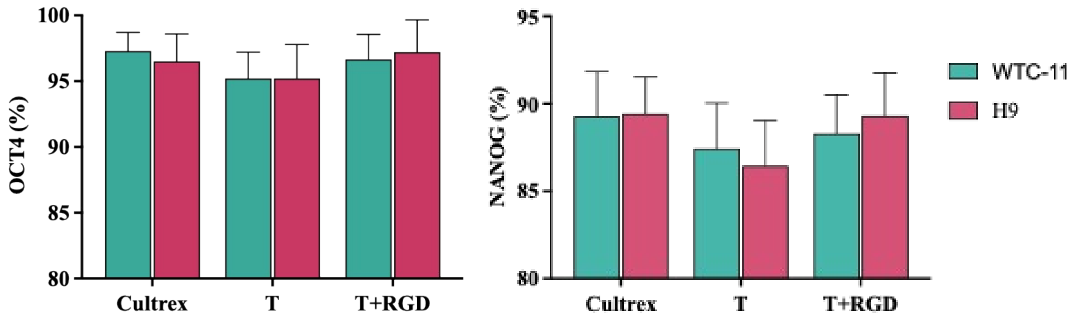

Supplementary Figure 1. Flow cytometry analysis of pluripotency markers OCT4 and NANOG in human pluripotent stem cells at passage 6 following sequential culture on synthetic terpolymer scaffolds. WTC-11 and H9 cell lines were cultured on Cultrex (control), terpolymer alone (T), and terpolymer functionalized with RGD peptide (T+RGD) for six sequential passages. Both OCT4 and NANOG expression levels were quantified by flow cytometry, demonstrating maintenance of pluripotency markers comparable to commercial substrate controls. Data represent mean ± standard deviation (n=5).

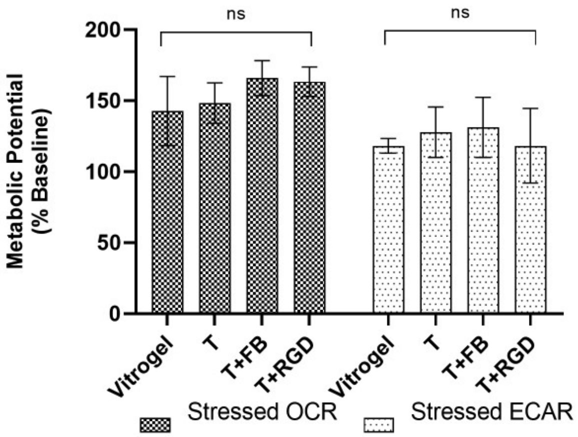

Supplementary Figure 2. Metabolic potential measured by Seahorse analysis showing stressed oxygen consumption rate (OCR) and extracellular acidification rate (ECAR) for WTC-1 encapsulated in 2:4:94 P400 terpolymer combination.

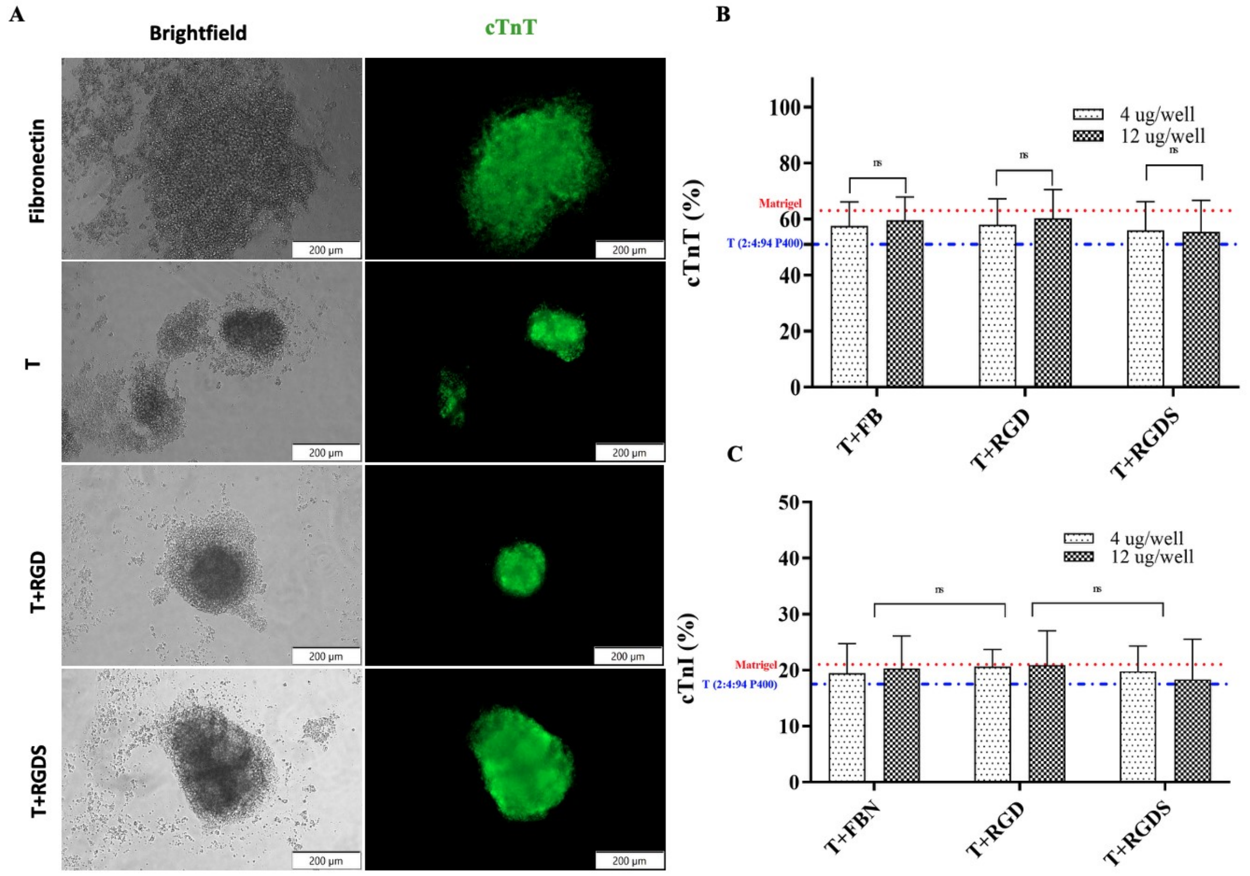

Supplementary Figure 3. Assessment of cardiac differentiation in cell spheroids cultured on various terpolymer in 2D. (A) Brightfield (left) and immunofluorescence images for cardiac troponin T (cTnT, green; right) of spheroids differentiated for 15 days on fibronectin, Terpolymer, T+RGD, or T+RGDS-coated. (B) Percentage of cTnT-positive cells relative to the total cell population, indicating the efficiency of cardiac differentiation. (C) Percentage of cTnT-positive cells normalized to the total spheroid area. Data are presented as mean ± SD (n = 4).

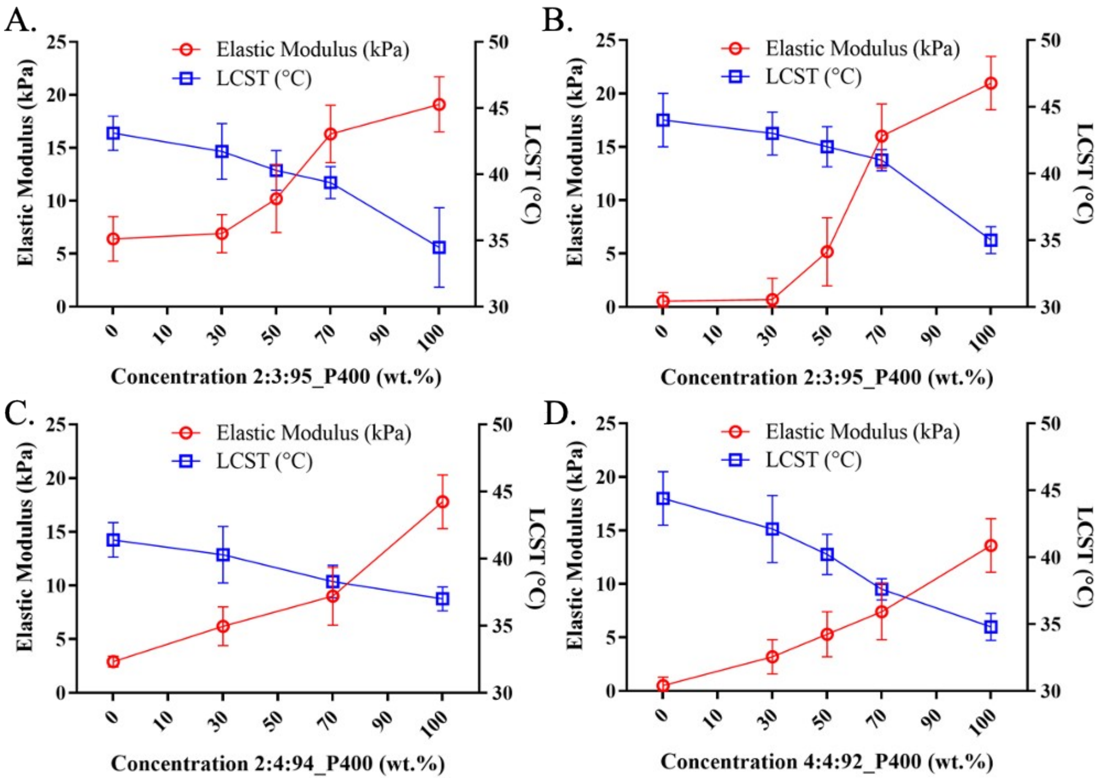

Supplementary Figure 4. Stiffness and LCST characterization of terpolymer blends. (A) Blend of 2:3:95 P400 with 10:4:86 P1000, (B) 2:4:94 P400 with 4:8:88 P400, (C) 2:3:95 P400 with 4:4:92 P1000, and (D) 4:4:92 P400 with 4:12:84 P1000. Each data point represents the average elastic modulus obtained at the corresponding polymer concentration. Measurements were conducted using an Anton Paar MCR302 rheometer.

Supplementary Table 1. Blends of terpolymers with distinct lower critical solution temperatures (LCST) and mechanical stiffness were formulated to investigate the combined effects of these properties within mixed systems. The LCST and elastic modulus of each individual terpolymer used in the blends are summarized in the accompanying table. All polymer mixtures were prepared at a final concentration of 15 wt.% in aqueous solution and subsequently analyzed using an Anton Paar MCR302 rheometer.

**Polymer Blend Combination**	**LCST (°C) Range (neat polymer)**		**Stiffness (kPa) Range (neat polymer)**	
2:3:95 P400 with 10:4:86 P1000	34.5 ± 0.9	43.1 ± 0.5	19.1 ± 3.3	6.4 ± 1.6
2:4:94 P400 with 4:8:88 P400	37.3 ± 0.4	40.6 ± 0.8	17.8 ± 2.5	2.9 ± 0.5
2:3:95 P400 with 4:4:92 P1000	42.8 ± 0.7	33.8 ± 0.7	0.5 ± 0.1	13.6 ± 1.3
4:4:92 P400 with 4:12:84 P1000	33.8 ± 0.7	44.1 ± 0.7	13.6 ± 1.3	0.5 ± 0.3

Supplementary Table 2. Stiffness and LCST of terpolymer blends exhibiting LCST values near physiological temperature (37 °C). The concentration column indicates the weight fractions of terpolymer 1 and terpolymer 2 within each blend. Following the mixing of both components, aqueous solutions were prepared at a final polymer concentration of 15 wt.% and subsequently characterized using an Anton Paar MCR302 rheometer.

Combination (Terpolymer 1 – Terpolymer 2)	Concentration (wt.%)	Stiffness (kPa)	LCST (° C)
2:3:95 P400 - 10:4:86 P1000	90 - 10	17.7	36.5
2:3:95 P400 - 4:4:92 P1000	90 - 10	18.5	37.5
4:4:92 P400 - 4:12:84 P1000	70 - 30	7.4	37.6
4:4:92 P400 - 4:12:84 P1000	90 - 10	11.5	35.7

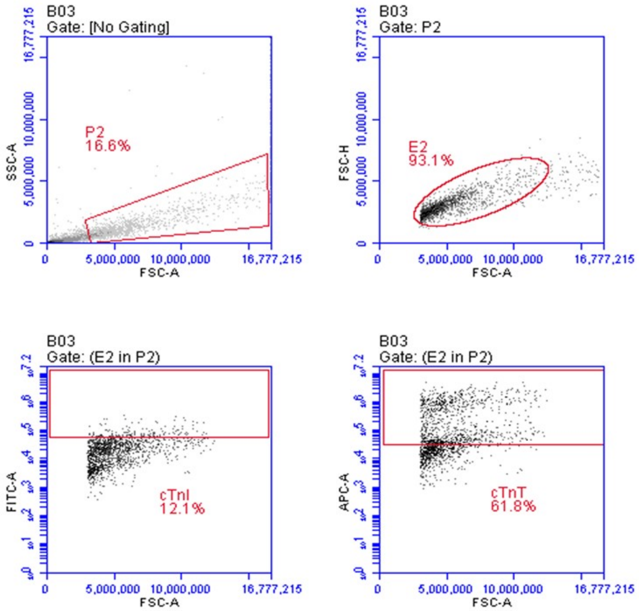

Supplementary Figure 5. Representative flow cytometry gating strategy for cardiac differentiation analysis. Flow cytometry plots showing the gating strategy used to quantify cardiac troponin I (cTnI) and cardiac troponin T (cTnT) expression in differentiated cardiomyocytes. Sequential gating was performed as follows: (top left) initial cell population without gating showing 16.6% viable cells in gate P2, (top right) live cell population (P2) representing 93.1% of gated events (E2), (bottom left) cTnI-positive cells within the live population showing 12.1% expression, and (bottom right) cTnT-positive cells within the live population showing 61.8% expression. Representative data from WTC-11 iPSCs differentiated for 15 days in terpolymer scaffold conditions. FSC-A: forward scatter area; SSC-A: side scatter area; FITC-A: fluorescein isothiocyanate area; APC-A: allophycocyanin area.
